# Only Making Things Worse: A Qualitative Study of the Impact of Wrongly Removing Disability Benefits from People with Mental Illness

**DOI:** 10.1007/s10597-016-0012-8

**Published:** 2016-05-19

**Authors:** Guy Shefer, Claire Henderson, Mary Frost-Gaskin, Richard Pacitti

**Affiliations:** 1 Health Service and Population Research Department, Institute of Psychiatry, Psychology and Neuroscience, King’s College London, De Crespigny Park, London, SE5 8AF UK; 2 Mind in Croydon, 26 Pampisford Road, Purley, Surrey, CR8 2NE UK

**Keywords:** Benefits, Disability, Disempowerment, Mental health, Welfare policy, Wellbeing

## Abstract

Many countries belonging to the Organisation for Economic Co-operation and Development (OECD) have seen a considerable increase in the number of disability benefits recipients (DBRs), in addition to an increase in the proportion of people with mental illness. As in other countries, changes to the welfare benefits system in England were made in order to reduce the number of DBRs. Many people lost their benefit payments, although a considerable number had them reinstated after appeal. Our aim was to investigate the impact of the process on DBRs whose disability was related to mental health and who won their appeal. Seventeen DBRs were interviewed. The participants reported three main types of impact. Beyond the practical reduction of income and the related anxiety, interviewees reported considerable stress when coping with the ‘never-ending’ cycle of bureaucracy. They also expressed anger, frustration and demoralisation at mistrust on the part of the authorities partly due to the ‘invisibility’ of their disability.

## Introduction

Over the last 50 years, many developed countries have witnessed dramatic increases in the rate of disability benefit awards. For example, data about long-term trends in disability benefit award rates from 11 countries which are members of the Organisation for Economic Co-operation and Development (OECD) in three continents, show that in all of them award rates increased between 1970 and 2008, and in six of them the rate had at least doubled during this period (OECD 2010). The data suggest that in many of these countries the main increase occurred in the two decades between 1970 and 1990 although, according to the same report, 15 out of 28 OECD member states still experienced considerable or slight increases in disability award rates between the years 1990 and 2008 (OECD 2010). The overall rising trend was common both to countries with very generous and liberal welfare policies such as Sweden (OECD 2010; Ulmestig 2013) and also to countries with much more stringent disability benefits systems such as the US (OECD 2010; Duggan 2006; Lindsay and Houston 2013). This consistent rise in disability benefit award rates and the accompanying growth in disability benefit expenditure became what is known in many countries as the ‘disability benefit crisis’ and has led to the development of policies aiming to tighten the assessment process, narrow the eligibility criteria, and reduce the amounts of money paid for disability benefits (OECD 2010; Duggan 2006; Lindsay and Houston 2013; Ulmestig 2013; Lunt and Horsfall 2013; van Berkel 2013).

Another, more recent trend regarding disability benefits has been an increase in the proportion of people with mental illness claiming disability benefits. In most OECD countries this rose from about 15–25 % in the mid 1990s to 30–50 % in 2009/10 (OECD 2012), so during that time it became the leading reason for claiming benefits in virtually all OECD countries (OECD 2010). This means that more than members of any other disabled group, people with mental illness felt the impact of the above restriction policies.

Over the years, a debate has developed about the extent to which those policies have been successful in returning people to long-term paid work (OECD 2010; Lindsay and Houston 2013; Lunt and Horsfall 2013; van Berkel 2013; Beatty et al. 2013; Brussig and Knuth 2013; Patrick 2011a). In contrast, relatively little has been said about their impact on the wellbeing and everyday life of claimants whose disability had prevented them from working. In this paper we hope to fill the gap, by reporting findings from a qualitative study of 17 disability benefit recipients with mental illness in London whose benefit claim was initially rejected but reinstated following an appeal.

### The Benefits Crisis in the UK

In line with trends in other countries, the number of incapacity benefit recipients in the UK increased considerably over the last three and a half decades, rising to 2.7 million in 2002, which was more than three times the number in the late 1970s (Weston 2012). In response, UK governments over the last 15 years have focused much of their welfare policy on attempts to reduce the number of recipients of disability benefit. Various regulations and schemes were developed in order to ‘activate’ individuals and move them from benefit dependency to paid employment. Initial attempts focused on more voluntary ‘support and advice’ schemes, but over the years the focus shifted to more direct attempts to tighten the benefit system (Weston 2012). These included, among other measures, the introduction of a new disability benefit, namely the Employment and Support Allowance (ESA). Eligibility for receiving the ESA was determined by the Work Capability Assessment (WCA) procedure, a new assessment protocol which replaced the less stringent Personal Capability Assessment (PCA).

More recently, in order to reduce the number of long-term disability benefit recipients as part of an overall aim to cut £18 billion from the welfare budget, the government decided that the WCA would be used to assess not only new ESA applicants but also all of those 1.5 million claimants whose eligibility for benefit had been approved in the past. It was anticipated that the change would lead to one in four of this group being found fit for work (Department for Work and Pensions 2010).

The new policy was a target for criticism and protest. Several academics and practitioners warned against its ‘conditional’ nature (Lindsay and Houston 2013; Patrick 2011a; b; Macnicol 2013; Grover and Piggott 2013) and against the growing labelling and blaming rhetoric accompanying it (Patrick 2011a; Turner 2011; Garthwaite 2014). However, most of the controversy surrounding it, was focused not on its rationale or rhetoric but on its implementation and specifically on the assessment process, the WCA—the operation of which was contracted out to the private firm Atos Healthcare. Following reports in the press about people who were terminally ill or severely disabled but nevertheless found fit for work, parliamentary committees, general practitioners, individuals and charities argued that the process was flawed and that it frequently made inaccurate assessments (Citizens Advice Bureau 2010); that it failed far too many people and unduly penalised people with specific health problems (Public accounts committee 2013); that the procedure was protracted, complex and stressful for claimants (Work and Pensions Committee 2014); and that it was degrading and dehumanizing (White 2013) or ‘brutal’ (Pilkington 2014).

Significantly, a high proportion of ‘fit to work’ decisions were appealed, and a high proportion of those appeals were successful. As at December 2014, 40 % of all ‘fit to work’ decisions from 2008 had been appealed (Department for Work and Pensions 2014). According to the same report, the rates of successful appeals varied over the years, ranging from 40 % in 2008 to 30 % in 2014. Disability benefits were wrongly removed from more than 114,000 people between October 2008 and February 2012 alone and this figure represents only the number of claimants who were being assessed for the first time ( Department for Work and Pensions 2013). It is not an exaggeration, therefore, to claim that over the whole period disability benefits were being wrongly denied to hundreds of thousands of people.

As in other OECD countries, a recent survey among disability benefit recipients found that 40 % of female disability benefit claimants and more than 30 % of male claimants report mental or behavioural problems as the main medical basis for their claim (Beatty and Fothergill 2013). Although these people constitute the largest group of ESA claimants, independent reports have stressed the unsuitability of the WCA for assessing people with mental illness (Litchfield 2013), and in one case the complaints were endorsed by a court ruling that the process substantially disadvantaged people with mental illness (Gentleman 2013).

While several qualitative studies had been conducted with benefit recipients in the UK in which their attitudes towards some of these policies were discussed (Weston 2012; Garthwaite 2014; Corden and Nice 2006; Patrick 2014), none of those studies focused either on people with mental illness or on people whose benefits had been wrongly stopped or reduced. By focusing on people whose benefits have been removed and subsequently reinstated, we aim to demonstrate the heavy human cost of the assessment and loss of benefits on claimants with mental illness and the ways in which those policy changes contributed to their social exclusion.

## Methods

The study is one element in a larger mixed-methods research project. As part of the quantitative element of the study, standardised measures of mental distress and use of health and social services were completed by two groups of people with mental illness: (1) those previously in contact with the benefits advice service run by a local branch of the national charity Mind, and who are currently in receipt of the correct benefits; and (2) those currently in receipt of help from this service in order to reinstate or avoid discontinuation of their correct benefit entitlement. For the qualitative study we sought to interview up to 20 service-users who were all members of the second group. Participants were recruited from this group using stratified purposive sampling so as to ensure diversity with respect to age, gender and ethnicity.

The interviews took place between October 2013 and May 2014, most of them at the offices of Mind in Croydon although one interview took place at the participant’s home. They were conducted by GS and lasted between 30 and 45 minutes; they were recorded and transcribed verbatim. Participants were asked about their benefits history, the specific problems for which they sought the advice service’s help and the impact, if any, of the processes regarding their benefits on their everyday life and on their mental health.

Analysis was thematic and involved four stages: (1) familiarization with the data and immersion in the data, including reading transcripts and notes and listening to the audio dialogue in order to extract the main themes and ideas; (2) thematic framework development, identifying the key issues and concepts present in the data and creating a coding tree, both inductively (based on the data) and deductively (based on the research questions), in which people’s views, experiences and behaviours could be organised; (3) indexing the data, i.e. grouping all data on the same theme; (4) interpretation, i.e. reviewing, making sense of the data, making typologies, and mapping the different ways in which the data are inter-connected (Spencer et al. 2013).

The study was approved by the Psychiatry, Nursing and Midwifery Research Ethics committee of King’s college London. The authors declare that there are no conflicts of interest. The funders had no role in study design, data collection and analysis, decision to publish, or preparation of the manuscript.

## Results

### The Sample

We interviewed 17 service users. Seven of the participants were male and ten female. There was a wide age-range, with younger participants in their early thirties and older ones nearing retirement age. Ten were white British, one was Black British, one of Black Caribbean origin, two of South Asian origin and one Polish. Two people provided no details about ethnicity. Mental illness diagnoses included schizophrenia, anxiety, depression, bi-polar disorder, paranoia, or a diagnostic combination. Seven of them also experienced physical problems affecting their mobility or ability to work for long hours. The benefits involved were the basic disability benefit—either ESA or its predecessor, Incapacity Benefit. In addition, people discussed the removal of other benefits such as the Disability Living Allowance (DLA), Housing Benefit and Income Support. Ten of the participants contacted the advice service for help to reinstate a benefit that had been stopped or reduced in value following reassessment. Four other cases involved appeal against a decision to deny a new benefit for which the participant had applied following a deterioration in their mental health. In one case, the appeal was made not against a reduction in benefit but against a requirement to take part in a work-related scheme. In the remaining two cases, in addition to removing existing benefits the authorities also demanded that the participants repay large sums of money.

The sums of money that were removed, reduced or demanded from the participants ranged from about £100 a month to about £100 a week. In some cases, this meant an increased rent contribution from the participant, as their Housing Benefit entitlement was reduced. With help from the benefits service advisors, all participants were eventually successful in their appeals. For most participants the process of submitting applications, taking part in assessments and making a succession of appeals was lengthy and might last more than a year. In 10 cases, the participants had to make more than one appeal before the initial decision could be overturned. At the time of the interviews, one participant had recently started working again in a part-time job after his physical condition improved; two did some voluntary work, but the others were not working.

### The Impact of Being Subject to Denial or Reduction of Benefit

#### The Impact of the Reduction of Income

For all participants whose benefits had been stopped or reduced this was a momentous and stressful event, with a range of consequences in terms of its impact on everyday life. Three participants lived with other family members who made some contribution to their income and thus shared the burden of reduction. For two participants, the period in which they waited for the appeal to be determined was relatively short and so the impact of the reduction was small. As noted above, one participant appealed not against a reduction in income but against a requirement to take part in work-related activity.

For the other participants, the impact of the financial reduction was much harder to bear. One of the participants, Hilary,^1^ was notified about stopping the payment just two weeks before Christmas. As she had neither savings nor any other income, she had to ask her friends for money to buy food:

They stopped paying my rent and don’t give me anything to eat during the Christmas. I didn’t get anything; people had to give me handout. […]I didn’t have anything. It’s people hand out to me. If people didn’t hand out for me, I wouldn’t have anything to eat. And I have to eat to take the medication.

Two other participants had realistic fears that they would have to leave their accommodation and be turned out onto the street because of the reduction. They were certain that they were able to stay in their houses only because of prompt intervention by the welfare benefit advisers. Another participant had savings but was forced to draw on them until they ran out, neither knowing whether the appeal would take place before the savings ran out nor knowing what to do if the appeal should fail. Brian, another participant, had to take extreme measures in order to survive on very little money:

If you can’t eat three meals a day, you’d have one meal a day and it’s not good for your health. There’s certainly no chance of socialising, absolutely no chance.

Brian further explained that even with the benefits, he was not able to live any kind of luxurious life but just to tried survive. With the cutting of benefits this task became harder:

When you’re already living on a small amount, you’ve already constrained yourself in a way someone who has an average or above average income wouldn’t understand. So it kind of takes you down to another level where you’ve just got to keep reducing yourself as much as you can and then in the end, if you keep reducing down, people are cutting back on things which are important for their health. Food and nutrition and exercise.

After her ESA claim was rejected, Rita had to ask her mother to draw on the small amount of money that her recently deceased grandmother had left to them. The money allowed her to buy food, but only until the appeal was heard.

The worries about the money and the need to ask friends for help was extremely stressful for these participants. They used terms such as ‘shocked’ ‘distraught’, ‘distressed’, ‘angry’, ‘devastated’, ‘struggled badly’, ‘nerve-racking’, and ‘suffering’ to describe their reaction to the loss of income or to their worries about its consequences. Two people reported that they had to ask friends for money or that the fear of being made homeless led them to think of suicide. Keith, who had to deal at the same time with the benefit reduction and a considerable rise in his rent explained:

Since I had my own place and it being threatened to being taken away from me it just made me more ill. I lost about 3 stone in weight, I was really, really ill down to the worry of not being able to meet the bills, not being able to eat properly.

#### The Stress Involved in Being Trapped in Cycle of Assessments, Rejections and Appeals

Loss of income, or the prospect of that loss, was not the only source of stress caused by the assessment process. Most participants reported how they struggled to cope with all the bureaucracy involved in what they described as an endless cycle of assessment, rejection and appeal. Most had made two or three appeals relating to the same benefit request, and by the time they won the last appeal most of them had only weeks or months or, at best, a year left, before they had to begin the same process all over again. One participant had completed three full cycles of assessment, rejection and appeal by the time of the interview, while others had completed two or were in the middle of the second cycle.

One of the reasons that this bureaucracy created so much stress was the time and effort people required to complete all the paperwork and their lack of skills for doing it. One participant remarked that it took him about a week to complete a single form. Others said they did not open official post because they were too anxious so they asked someone else to open it for them. Some participants noted that the questions were misleadingly worded and that, following past experience where their answers were distorted or assigned a different meaning from what they had intended, the task of answering the questions was particularly challenging. As Ron said, ‘It’s the way they word the questions. I don’t know what they are looking for, but they obviously know what they are looking for’. All participants, without exception, insisted that because of the stress involved and because the procedures were not suited to assess the eligibility of people with mental illness, they could not and would not pursue the proceedings without the help of the advice service.

A particular source of stress was the requirement to appear in person before some of the assessment panels or appeal tribunals. Some participants found the experience daunting. Rozlyn recalled how she became so anxious in one of the medical assessments that ‘I couldn’t speak, I was crying, I was hysterical’, and she ‘almost called an ambulance’. Margaret recounted how the very formal nature of one of the appeal hearings that she attended, the inquisitorial nature of the process, and the unsympathetic judge, all made the process ‘very scary and intimidating’. Rita’s stress caused by having to appear in front of the tribunal was caused by her anger of feeling she is treated as a liar (see further below) and the stress involved in talking about and re-living traumatic experiences in her life especially as she felt panel members were indifferent and did not care:

You are sitting there talking to them and you can see they are not listening to you. Hello, I am talking to you about something that is really intimate about me. And they are like zombies and then tick that box because they get commission or something for getting people off it so there you go, they are thinking about lining their pockets aren’t they? Which makes me angry.

Margaret explained:

I was sick with worry. I was really, really bad that time because I just thought I can’t go through this again. It was just no, not again, I’ve already been through it twice. I had also DLA. I went through and failed DLA, so I had recently been through DLA appeal and then a month later I got called for an ESA appeal.

Beth told how the cycles of bureaucracy affected her attempts of recovery:

It is like them picking at a scab. There are times actually in my life since I’ve got here and I’ve thought, Yeah, I am actually getting somewhere, I have made it to the local shop on my own. I’ve made these steps on my own, sometimes I just think just leave me alone so I can get better, so I can help myself. But then they’ll come along and they’ll pick and say you’ve gotta come to this or you’ve got to come to that, you’ve got to be here or we’re stopping your money. And you are just like oh my god, and then I just go in my kitchen and sit on the floor and sob. You might have took 5 baby steps but it throw you 10 back.

*Interviewer*: Why?

Because it causes so much stress and anxiety that it will just not leave your mind. You’ve got to do this, you’ve got to go to this thing, you’ve got to see these people. Before you go there [the medical assessment panel] it’s a fear.

#### The Invisibility of the Disability and the Anger About Being Mistrusted

Some of the participants were offended by, or felt angry about, the messages implied in the rejection of their claim, as if they were not telling the truth or were applying for money to which they were not entitled. Related to this was the frustration experienced by other participants, who struggled to find ways to prove or demonstrate why they were unable to work, given that their disability was not physical and therefore not always visible. The view among many participants was that the assessment procedure was more suitable to assess physical disability than it was to assess mental health. Two participants said that the frustration about the invisibility of their disability led them to wonder if they should make an effort to appear more like the negative stereotype of someone with mental illness in terms of self-presentation, in order to convince the committee that their case was genuine. Even the thought of having to appear less presentable in order to convince the committee was upsetting and frustrating.

Deborah reported how a member of the panel assessing her eligibility suspected she was lying to them. Specifically this doctor suggested she did not need a walking stick, that she wasn’t using it properly and that she was not left handed as she appeared to be. She found this suggestion highly offensive:

I mean, please, I know whether I am left-handed or not. I was born in an era whereby you weren’t allowed to be left-handed, so I write with my right hand because I had to sit on my left hand. But certain things I cannot do with my right hand.

The fact that they were mistrusted was particularly painful for participants who had previously lived through a period when people close to them did not believe them or accused them of lying. This made the feeling of being mistrusted by the authorities even more traumatic. Rita explained:

My doctor called me a liar, my sister, my mum called me a liar.

So you are writing down this form and they have read that form but why are they saying no, you must come to an assessment, we don’t believe you. It’s like saying we don’t believe you, you are calling me a liar. That’s what I’ve been called by kids at school, you know don’t call me a liar, I am not a liar, why would I lie? So that is what is hard, straight away people sit there judging you and why are you judging me? Don’t call me a liar, this is difficult enough without that.

Ashley talked about how stressed he felt because of the process ‘almost makes you feel guilty for being disabled’. Margaret described how she was frustrated by the formal and alienating nature of the process and the predisposed disregarding and non-believing attitude towards the individual applicant:

‘I am not a number, I am an individual, I am a person. You feel as though when you’ve been dealt with that you’re just another person…a number. You never feel as though they are on your side, that’s what it is. When they are reading [the forms completed by the applicant] you are not sure they are really taking in what you are saying and it’s only when you’ve got an organisation or a mental health team on your side that they will listen to what you have to say.’

As in other studies (Corden and Nice 2006), even though they were not asked about it, eight of the participants explicitly accepted the assertion that some people are trying to cheat the system by submitting false claims and pretending to be disabled even though they are not. These participants perceived the wish of the government to try and stop fraudulent application as legitimate and were aware of the challenges involved in doing so. However, they did not think this aim justified treating all the people who receive benefits as suspects. In addition, and again, without being asked about it, some of the participants stressed how much they would have liked to be able to work, and argued that those who believed they would rather receive benefits than go to work did not understand people with mental illness. People who had previously been in employment emphasised the drastic reduction in income and, as a result, the corresponding reduction in their quality of life when coming to live on benefits. This point was made in order to affirm that they would never have chosen to live on benefits if they could go to work. Others also stressed both the self-stigma and stigmatising attitudes towards them as further reasons why they would have preferred to work if only they could. Margaret said she was ashamed that she was receiving benefits and felt like a ‘benefit scrounger’, but given her anxiety she was unable to leave the house or get on a bus, let alone go to work. Some of the participants expressed optimism and determination that in the future they would indeed go back to work. However, as we noted above, some felt that the constant cycle of benefits assessments and rejections made their attempts to recover much more difficult.

## Discussion

There is an undisputed need for any benefits system to verify that people who receive benefits have genuine disabilities that do not allow them to work. Moreover, it is unrealistic to expect governments to make no attempts to reduce the number of disability benefit recipients, particularly in view of an increasing rate of benefit recipients in recent decades. However, the debate about the most effective way to reduce fraud or increase the ability of disabled people to engage in long-term paid work should not detract from the considerable harm caused by the assessment process to those people who are clearly unable to work. Denying benefits to people with genuine mental health disability is one of the most severe forms of social exclusion. Those who advocate tightening the assessment procedures may rely on the existence of the appeal system as a form of safety net to ensure that people who suffer from genuine disability will continue to receive benefits. However, for claimants with mental illness the rejection of their claim and the ensuing appeal process is far from being a mere correction to an unfortunate technical error. For them, the process is loaded with endless frightening challenges and its outcome is far from obvious or predictable.

One of the most worrying, albeit expected, findings from the interviews is that people who are unable to work are left without basic subsistence for considerable periods of time until their appeals are heard. This puts some of them in desperate situations where they need to rely on help from friends in order to provide for their basic needs. However, this is not the only negative impact of current practices. The constant cycle of assessment, claim rejection, appeal and new assessment and claim rejection bring constant stress and anxiety to people whose lives are already full of struggles. It adds anger and frustration and increases self-stigma and a sense of helplessness. In many cases, after successfully travelling a long and winding road, they have to begin the process all over again. This only reinforces the view that the aim is to make the life much harder for all benefit claimants, even the genuine ones.

The process also creates special challenges for people with mental illness in terms of its impact. For people who struggle with some kind of mental illness, the stress involved in dealing with the many forms, constant demands for re-assessment, and the need to appear in person on a regular basis before tribunals can be extremely unsettling and anxiety-provoking (although it must also be a considerable challenge for people who do not suffer from mental illness). Likewise, the need to repeatedly re-state one’s case and prove entitlement to benefit can be frustrating for anyone, but it can be particularly stressful for people who see the implication that they are lying as a personal attack on their integrity (rather than as a formal procedure which all benefit claimers have to undergo), and in some cases this can bring back previous traumatic experiences. Denying people with mental illness the basic income to which they are entitled is not only unfair and cruel; it can also be counterproductive. Most interviewees described the stress involved in this process as a serious threat to their mental health. Worse mental health does not only mean a lesser likelihood of coming off benefits; it can also result in additional public costs from increased use of the health service.

The harshness of these consequences could be minimised if policymakers were to pay greater consideration to the particular difficulties of people with mental illness; to differences in the nature of disabilities; to differences in ascertainment and confirmation between people with physical disability and people with mental disability; and, not least, to the detrimental impact of the constant re-assessment cycle. The quality and availability of free benefits advisers can also make a huge difference. For example, while the benefits advisers to participants in this study are working in a branch of a large national charity, as far as they are aware theirs is the only local branch which provides a benefits consultancy and support at that level.

### Limitations

As with most qualitative studies, the sample size is small and cannot be considered statistically representative. It is also limited to a restricted geographical area in the UK. There is a need for larger-scale quantitative and qualitative studies in the UK and other countries, in order to depict the overall picture of the impact of disability benefits policies on people with disabilities related to mental illness.

## Footnotes

1 All the names used here are pseudonyms to prevent identification.
